# Transcriptomic analysis of primary nasal epithelial cells reveals altered interferon signalling in preterm birth survivors at one year of age

**DOI:** 10.3389/fcell.2024.1399005

**Published:** 2024-07-24

**Authors:** Denby J. Evans, Jessica K. Hillas, Thomas Iosifidis, Shannon J. Simpson, Anthony Kicic, Patricia Agudelo-Romero

**Affiliations:** ^1^ Wal-yan Respiratory Research Centre, Telethon Kids Institute, Nedlands, WA, Australia; ^2^ Wesfarmers Centre of Vaccines and Infectious Diseases, Telethon Kids Institute and The University of Western Australia, Crawley, WA, Australia; ^3^ School of Population Health, Curtin University, Bentley, WA, Australia; ^4^ Centre for Cell Therapy and Regenerative Medicine, School of Medicine and Pharmacology, The University of Western Australia and Harry Perkins Institute of Medical Research, Nedlands, WA, Australia; ^5^ School of Allied Health, Curtin University, Bentley, WA, Australia; ^6^ Department of Respiratory and Sleep Medicine, Perth Children’s Hospital, Nedlands, WA, Australia; ^7^ School of Molecular Science, University of Western Australia, Nedlands, WA, Australia; ^8^ European Virus Bioinformatics Centre, Jena, Thuringia, Germany

**Keywords:** preterm, airway epithelial, RNA seq analysis, interferon, bronchopulmonary dysplasia

## Abstract

**Introduction:** Many survivors of preterm birth (<37 weeks gestation) have lifelong respiratory deficits, the drivers of which remain unknown. Influencers of pathophysiological outcomes are often detectable at the gene level and pinpointing these differences can help guide targeted research and interventions. This study provides the first transcriptomic analysis of primary nasal airway epithelial cells in survivors of preterm birth at approximately 1 year of age.

**Methods:** Nasal airway epithelial brushings were collected, and primary cell cultures established from term (>37 weeks gestation) and very preterm participants (≤32 weeks gestation). *Ex vivo* RNA was collected from brushings with sufficient cell numbers and *in vitro* RNA was extracted from cultured cells, with bulk RNA sequencing performed on both the sample types. Differential gene expression was assessed using the limma-trend pipeline and pathway enrichment identified using Reactome and GO analysis. To corroborate gene expression data, cytokine concentrations were measured in cell culture supernatant.

**Results:** Transcriptomic analysis to compare term and preterm cells revealed 2,321 genes differentially expressed in *ex vivo* samples and 865 genes differentially expressed in cultured basal cell samples. Over one third of differentially expressed genes were related to host immunity, with interferon signalling pathways dominating the pathway enrichment analysis and *IRF1* identified as a hub gene. Corroboration of disrupted interferon release showed that concentrations of IFN-α2 were below measurable limits in term samples but elevated in preterm samples [19.4 (76.7) pg/ml/µg protein, *p* = 0.03]. IFN-γ production was significantly higher in preterm samples [3.3 (1.5) vs. 9.4 (17.7) pg/ml/µg protein; *p* = 0.01] as was IFN-β [7.8 (2.5) vs. 13.6 (19.5) pg/ml/µg protein, *p* = 0.01].

**Conclusion:** Host immunity may be compromised in the preterm nasal airway epithelium in early life. Altered immune responses may lead to cycles of repeated infections, causing persistent inflammation and tissue damage which can have significant impacts on long-term respiratory function.

## Introduction

Poor respiratory outcomes are well documented in those born preterm, before 37 weeks completed gestation. Symptoms such as cough and wheeze are widely reported throughout infancy and childhood (Greenough, 2008; Pramana et al., 2011; Vrijlandt et al., 2013) and approximately 60% of those born very preterm (≤32 weeks gestation) will be re-hospitalised for respiratory causes in the first 3 years of life regardless of if they had a neonatal diagnosis of the chronic lung disease bronchopulmonary dysplasia (BPD) (Hong et al., 2016). In addition to persistent respiratory symptoms, reduced and declining lung function is reported in those born preterm (Doyle et al., 2017; Um-Bergström et al., 2017; Simpson et al., 2018). One proposed mechanism contributing to preterm associated lung disease is alterations within the airway epithelial cells (Looi et al., 2019). The airway epithelium acts as a physical barrier and orchestrator between the external environment and the lung milieu, providing the first line of defence against foreign pathogens and infection (Schleimer et al., 2007; Whitsett and Alenghat, 2015). Disruptions within the airway epithelium can lead to long-term consequences on lung health and is altered in other respiratory diseases including asthma (Kuperman et al., 2002; Wark et al., 2005; Kicic et al., 2006), cystic fibrosis (Frizzell et al., 1986; Sutanto et al., 2011) and chronic obstructive pulmonary disease (Patel et al., 2003; Comer et al., 2013). The extent to which the epithelium may be disrupted in those born preterm remains largely unknown, highlighting the need for further research in this area.

Whilst the contribution of the airway epithelium to preterm lung disease is not fully understood, an altered transcriptomic signature has previously been identified in basal airway epithelial cells of preterm-born infants in the initial minutes following birth (Shui et al., 2021). These data suggest that preterm delivery disrupts the normal development of the airway epithelium. However, the transcriptomic profile has not been examined beyond the neonatal stage in those born preterm. It is consequently difficult to delineate if the altered transcriptomic signature observed immediately after birth represents acute changes that will resolve over time, or if preterm birth survivors have a distinct transcriptomic signature detectable within the airway epithelium. If an altered transcriptomic signature also exists after the first year of life, it is possible that these changes may provide valuable insight into the underlying mechanisms driving ongoing respiratory symptoms within this population.

In this study, nasal airway epithelial samples obtained after the first year of life were used to test the hypothesis that distinct transcriptomic profiles were detectable within the nasal airway epithelium of preterm birth survivors outside of a neonatal intensive care setting. Specifically, we aimed to identify any transcriptomic differences between *ex vivo* nasal airway epithelial cells collected from term and very preterm individuals and whether these were retained in an *in vitro* cell culture model of the preterm nasal airway epithelium.

## Methods

### Participants and clinical data

Sampling in preterm participants occurred while participants were under sedation during a research appointment to assess lung function at the Princess Margaret Hospital for Children (now Perth Childrens Hospital, Perth, Australia) as part of the Preterm Infant Function and Clinical Outcomes (PIFCO) cohort study (Freislich et al., 2022; Stoecklin et al., 2023; Urs et al., 2023). Preterm participants with and without a diagnosis of BPD were included. Bronchopulmonary dysplasia was defined as the requirement for 28 days of supplemental oxygen as assessed at 36 weeks postmenstrual age (Jobe and Bancalari, 2001). Term infants with no prior history of respiratory disease were sampled during elective surgery (as part of the Western Australian Epithelial Research Program) (Iosifidis et al., 2020; Kicic et al., 2020). Both term and preterm participants’ samples were used to establish *in vitro* cell cultures. All participants were asymptomatic for respiratory tract infection at the time of sampling. Ethics approval was granted by each institution’s human ethics committees with informed written consent provided by a participants’ parent or guardian (Child and Adolescent Health Service #2014083EP, St John of God Subiaco #901.1421 and Curtin University #HRE 2021-0489 and #HRE 2019-0086).

### Sample collection and cell culture

Nasal airway epithelial cells were collected via brushing of the nasal turbinate of each nostril using two 2.0 mm cytology brushes (Olympus, Tokyo, Japan). These were then placed into transport medium and processed within 30 min after collection as described (Kicic et al., 2020). Briefly, cells were initially vortexed off the cytology brushes following which the brushes were then removed. The cell suspension was then gently passed consecutively through a 27G and 25G needle to form a single cell suspension. A cell count was performed using Trypan Blue staining and if cell number permitted, 1 × 10^6^ cells fractioned and resuspended in 350 µL RLT lysis buffer containing 1% (v/v) ß-mercaptoethanol (Qiagen, Hilden, Germany) for *ex vivo* RNA collection. To determine if transcriptomic differences were retained in cell culture, monolayer cell cultures were also established by seeding 125,000 primary cells in a 1:1 ratio with irradiated NIH-3T3 fibroblasts in fibronectin pre-coated tissue culture flasks as previously described (Martinovich et al., 2017). Once confluent, monolayers were trypsinised and 1 × 10^6^ cells re-suspended in 350 µL RLT lysis buffer containing 1% (v/v) ß-mercaptoethanol for RNA collection. All samples were stored at −80°C until recruitment was complete.

### RNA extraction and sequencing

Batch extraction of RNA was performed using the Ambion Purelink RNA mini kit (Thermo Scientific, Waltham, Mass) as per the manufacturer’s instructions and RNA integrity number (RIN) determined using an Agilent 2100 Bioanalyzer (Agilent Technologies, California, United States). High-quality RNA samples (average RIN 9.24 ± 0.99) were used to build TruSeq Illumina Stranded mRNA libraries (Illumina, California, United States) which were then sequenced (Illumina NovaSeq 6000; 100 bp Single-End reads) by the Australian Genome Research Facility (AGRF; Victoria, Australia).

### Bioinformatic analysis

Each sample library was split over two lanes within the same flow cell and sequenced. Raw FASTQ files of the same sample were concatenated containing on average 28.1 million (M) reads. The data were then processed by quality-control steps (adapter removing, filtering and trimming) using FastQC (https://www.bioinformatics.babraham.ac.uk/projects/fastqc/). High-quality reads were aligned to the human reference genome (ENCODE; male hg19) using Spliced Transcripts Alignment to a Reference (STAR; v2.7.10a) (Dobin et al., 2013). Human genome mapping percentage was evaluated with Samtools (v1.7) (Li et al., 2009) which ranged between 95.22% and 99.87%. Gene-level quantification of raw counts was then performed using RNA-Seq by Expectation Maximization (RSEM; v1.3.3) (Li and Dewey, 2011).

In addition, published *ex vivo* expression data from 14 children born at term (and sampled as part of WAERP) were used to supplement numbers in this study and formed the *ex vivo* term population (Kicic et al., 2020). Data were downloaded from the National Centre for Biotechnology Information’s (NCBI) Gene Expression Omnibus (GEO), (Accession number; GSE118761) with files corresponding to the nasal brushings from non-atopic non-wheezer children, i.e., SRR7721184; SRR7721186; SRR7721188; SRR7721190; SRR7721192; SRR7721194; SRR7721196; SRR7721198; SRR7721200; SRR7721202; SRR7721204; SRR7721206; SRR7721208; SRR7721210. Term *ex vivo* data was analysed under the same pipeline described above. Here, raw FASTQ files averaged 24.1 M reads and mapping percentage fluctuated between 99.86% and 99.91%.

### Differential expression analysis

Differential gene expression analysis was performed using the R package limma (version 3.54), where trend = TRUE (Law et al., 2014). Participant age and sex was built into the model and surrogate variable analysis was performed using the BatchQC package to remove further confounders (Manimaran et al., 2016). Genes were considered statistically different, after multiple testing adjustments using the Benjamini-Hochberg method, if False Discovery Rate (FDR) < 0.05 and Fold Change (FC) > 1.5 (Benjamini and Hochberg, 1995). Pathway enrichment and network analysis was performed on differentially expressed genes using the ReactomePA (version 1.42) and clusterProfiler (version 4.6.0) R packages in RStudio^®^ (version 4.2.2) (Yu et al., 2012; Yu and He, 2016). Gene interactions were examined by zero-order protein-protein interaction networks using network analyst https://www.networkanalyst.ca/which were constructed using the InnateDB database (Breuer et al., 2013). Cellular deconvolution was performed on *ex vivo* samples to estimate proportions of cell types within the collected samples using CIBERSORTx (Newman et al., 2019) and reference signatures from the human lung cell atlas (Sikkema et al., 2023).

### Corroboration of differentially expressed genes

To corroborate bulk RNA sequencing findings, concentrations of IFN-α2, IFN-β, and IFN-γ were measured in cultured cell supernatants from an independent sample set. Briefly, primary nasal airway epithelial cells were grown at air-liquid interface until confluent and 50 µL of supernatant sampled from both the apical and basolateral compartment as previously described (Martinovich et al., 2017). Cultured supernatant was run using a commercial multiplex assay (BioRad, California, United States) to determine the interferon concentration present in the sample. Detection limits for the assay prior to protein correction were as follows: IFN-α2: 28.5–62,267 pg/mL; IFN-β: 14.1–3,490 pg/mL; and IFN-γ: 13.7–2,860 pg/mL. All measured concentrations were normalised to the total protein content of each sample. Statistical significance was determined using the Mann-Whitney *U* test as appropriate for the non-parametric data distribution and variance. Samples that were below detection limits of the assay were presented as half of the lowest standard and protein corrected.

## Results

### Demographics

As part of the wider PIFCO project, we sampled 35 preterm infants (full cohort demographics provided in Supplementary Table S1) and of these, 10 had sufficient cell numbers at collection to facilitate RNA collection and were reserved for *ex vivo* RNA sequencing. However, one preterm sample did not subsequently pass sequencing library quality checks and was thus excluded from analysis. Cell cultures were successfully established and had sequencing performed from 11 preterm participants. However, only three of these overlapped with the sequenced *ex vivo* samples and most were independent participant samples. No overlap was present in the term *ex vivo* and *in vitro* samples. Demographic data for all samples utilised within this study are provided in Table 1.

**TABLE 1:** Demographic data for all participant samples included in each analysis type*.* Data presented as mean ± standard deviation or median [IQR], unless otherwise specified.

	*Ex vivo* samples		*In vitro* samples		Validation samples	
	Term	Preterm	Term	Preterm	Term	Preterm
Sample (n male)	12 (4)	9 (9)	8 (4)	11 (9)^a^	5 (2)	11 (9)
Age at Brushing (Actual Years)	3.73 ± 1.66	1.45 ± 0.10	3.04±1.09	1.40 ± 0.12	3.01 ± 0.39	1.34 ± 0.10
Gestational Age (Weeks)	>37	27.2 ± 2.81	>37	28.32 ± 1.95	>37	28.26 ± 2.53
Bronchopulmonary Dysplasia (%)	—	4 (44%)	—	4 (36%)	—	4 (36%)
Birthweight Z-Score	—	−0.33 ± 0.78	—	−0.12 ± 0.89	—	−0.43 ± 0.87
Total Hours Oxygen	—	61 [1597]	—	59 [1203]	—	198 [1261]
Total Hours Respiratory Support (Ventilation, CPAP and HHF)	—	1401 ± 895	—	1313 ± 729	—	1231 ± 770

CPAP, continuous positive airway pressure; HHF, humidified high flow.

^a^ Three of the preterm *ex vivo* samples were successfully cultured and used for *in vitro* analysis. There was no overlap in term participants.

### Differential expression analysis of e*x vivo* cell samples

**FIGURE 1:**
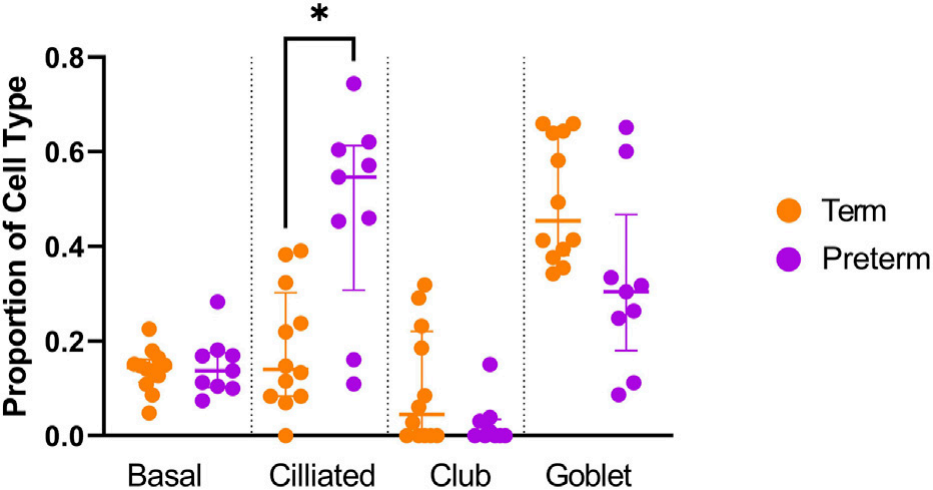
Cellular deconvolution analysis of *ex vivo* preterm nasal cell samples. The Human Lung Cell Atlas and CIBERSortx database were used to approximate the percentage of cell types within the *ex vivo* samples. A significantly higher (*p* < 0.05) proportion of ciliated cells was identified within the preterm cohort (n = 9) compared to the term cohort (n = 12).

**FIGURE 2:**
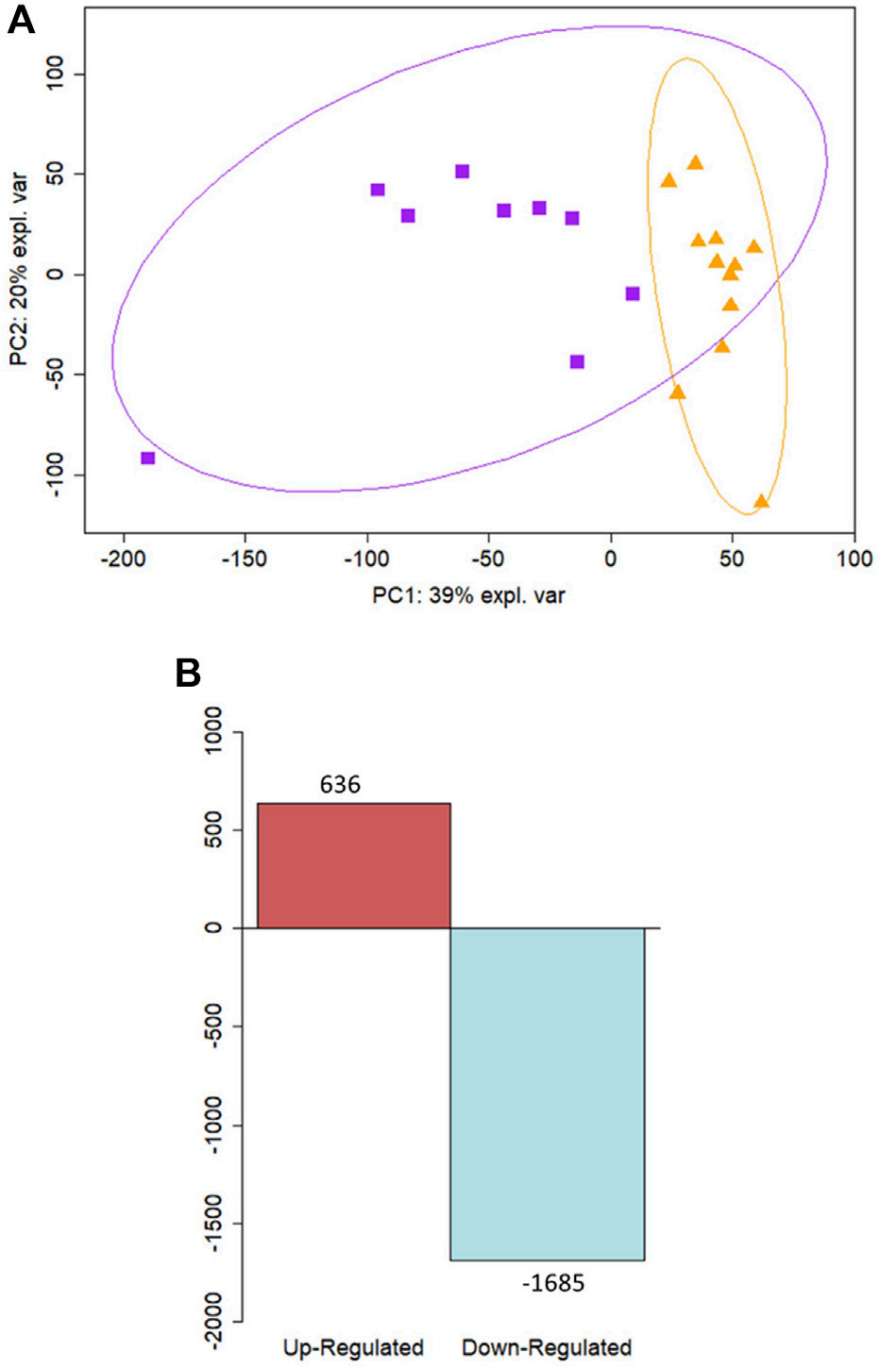
Differential expression analysis reveals distinct profiles between term and preterm *ex vivo* samples. **(A)** Principal component analysis of *ex vivo* samples illustrates a separation along the PC1 component between samples from preterm participants (n = 9) shown in purple squares and samples from term participants (n = 12) in orange triangles. **(B)** Differential expression analysis revealed 636 upregulated genes and 1685 downregulated in preterm *ex vivo* samples compared to term controls.

Cellular deconvolution was first performed to provide an estimate of cell types present within the *ex vivo* term and preterm samples. As expected, cellular deconvolution of the *ex vivo* samples identified primary cell types as ciliated, basal, club and goblet epithelial cells (Figure 1). Preterm samples showed a significantly larger proportion of ciliated cells compared to term (median [IQR]: 54 [31] vs 14 [29] %, *p* = 0.03) and lower, but not statistically significant, proportion of goblet cells (30 [29] vs 45 [28] %, *p* = 0.4). Comparative analysis of baseline transcriptomic profiles was then performed within the *ex vivo* samples which identified clear separation between term and preterm samples (Figure 2A). The top 10 genes identified as contributing maximally to the separation along the first component were *UBE2K*, *ACBD3*, *UBQLN1*, *TMEM87B*, *TMEM33*, *MARCHF5*, *PTBP3*, *MED13*, *SPCS3*, and *USP38*. Differential gene expression analysis identified 2,321 genes that were significantly different between the two populations. Of these, 636 genes were upregulated and 1,685 were downregulated (Figure 2B). The top 10 up and downregulated genes have been outlined in Table 2 and a full list of differentially expressed protein-coding genes is available (Supplementary File S1). The top 10 upregulated genes were all ribosomal proteins or pseudogenes, with the exception of the calcium channel gene *CACNG6*. The top 10 downregulated genes similarly contained multiple ribosomal proteins and pseudogenes, and additionally contained the T-cell activation marker *CD69*, glycoprotein receptor *CD200R1*, the enzyme *CYP1B1* and G protein regulator *RGS1*. Many of the remaining differentially expressed genes were identified as those relating to innate immunity through the InnateDB database, including genes such as *IL32*, *IRF1*, *TLR2*, *CASP7*, and *CD8A*.

**TABLE 2:** The top differentially expressed genes in *ex vivo* preterm samples compared to term. The top 10 up and downregulated genes (ranked by fold-change) are displayed.

Upregulated			Downregulated		
SYMBOL	log Fold Change	Adjusted *p*-value	SYMBOL	log Fold Change	Adjusted *p*-value
*RPL7*	5.81	<0.001	*RPL10P3*	−8.15	<0.001
*RPS27*	5.67	<0.001	*MTND4P12*	−6.07	<0.001
*PRKY*	5.66	0.048	*MTCO1P12*	−4.45	0.022
*RPL21*	5.32	<0.001	*CD200R1*	−3.61	<0.001
*RPS28*	4.31	<0.001	*CYP1B1*	−3.39	0.008
*RPS3A*	4.29	<0.001	*MS4A1*	−3.14	0.049
*RPL9*	4.29	<0.001	*CD69*	−3.11	0.001
*RPS26*	3.77	<0.001	*RGS1*	−2.97	0.005
*CACNG6*	3.75	0.019	*RNASE1*	−2.97	<0.001
*RPL18A*	3.57	<0.001	*SLC26A4*	−2.81	0.033

**FIGURE 3:**
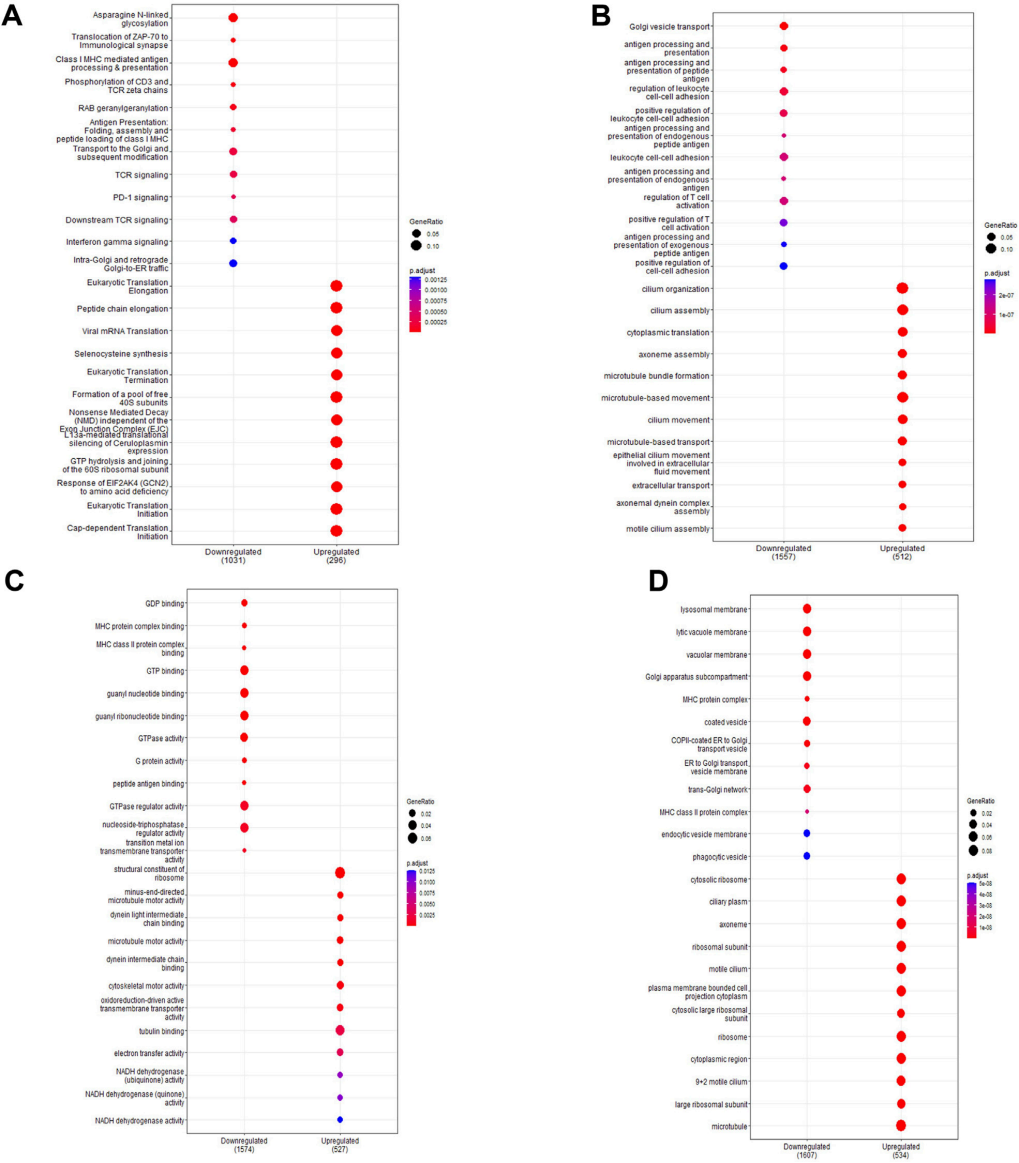
Pathway enrichment analysis of *ex vivo* samples. Pathway enrichment analysis was performed on differentially expressed genes detected in preterm samples (n = 9) compared to term (n = 12) using the Reactome and clusterProfiler packages. Enrichment was done using the Reactome database **(A)** and the Gene Ontology database for biological processes **(B)**, molecular function **(C)** and cell components **(D)**. The top 10 up and downregulated pathways are shown for each enrichment performed. The gene ratio (number of differentially expressed genes over the total pathway genes) is represented by the size of the circle, with the colour of the circle denoting the significance level of the *p*-value.

Pathway enrichment analysis highlighted which functional gene sets were enriched within the differentially expressed genes (Figure 3; full list in Supplementary File S2). Pathways were examined across the Reactome (Figure 3A) and Gene Ontology databases for biological processes (Figure 3B), molecular function (Figure 3C) and cell component (Figure 3D). Top enriched pathways occurring multiple times in downregulated genes included interferon signalling (Pathway IDs: R-HSA-877300, R-HSA-913531, GO:0034341, GO:0071346, GO:0032729, GO:0032609, GO:0032649), T-cell activation (GO:0042608, GO:0050863, GO:0050870, GO:0042098, GO:0046634, GO:0046631, GO:2000514, GO:2001185) and antigen processing (R-HAS-983169, R-HAS-983170, R-HAS-1236975, R-HSA-983168, GO:0019882, GO:0019882, GO:0002483, GO:0019883, GO:0002478, GO:0002503, GO:0019884, GO:0002501, GO:0019885, GO:0002474, GO:0019886, GO:0002504, GO:0002495, GO:0042605). Top enriched pathways in upregulated genes included eukaryotic translation (R-HSA-156842, R-HSA-72764, R-HSA-72613) and cilium assembly and movement (R-HSA-5617833, GO:0044782, GO:0060271, GO:0003341, GO:0003351, GO:0044458, GO:0001539, GO:0060285, GO:0042073, GO:0061512, GO:0060294, GO:0035721, GO:0060287, GO:0003356, GO:0097014, GO:0031514, GO:0097729, GO:0036064, GO:0097730, GO:0030990). Network analysis showed a large proportion of differentially expressed genes were immune-related (Supplementary Figure S1A), with further network interrogation identifying a cluster of genes relating to interferon signalling (Supplementary Figure S1B). Genes within the cluster were *CYLD*, *CAMK20*, *DDX21*, *IFNGR2*, *GFPT1*, *HLA-C*, *UBEK2K*, *HLA-B*, *GFPT1*, *B2M*, *HLA-C*, *HLA-G*, *JAK-2*, *CD8A*, *PSMA2*, *CAP2A1*, *CUL3*, *SRPK1*, *NUDT21*, *CAND1*, *SMARCE1*, *PRPF40A*, *TFG*, *BARD1*, *TAP1*, *CHUK*, *STAT1*, *IRF1*, *TFRC*, *COPB1*, *PPP1CC*, *CDK1*, *CDKN1B*, *ATM*, *SIRT1*, *CSNK2B*, *ETS1*, *PIK3CA*, *NR3C1*, *ZHX1*, *GPB2*, *BID*, *TLR2*, *CREB1*, *LCK*, *ATP1B1*, *HLA-DRB5*, *MS4A1*, *HLA-DRA*, *HLA-DRB1*, *RFXAP*, *HLA-DPA1*, *HLA-DPB1*, *HIF1A*, and *EZH2*. Hub genes included *IRF1*, *CD8A*, *HLA-B*, *CREB1*, *SIRT1*, *STAT1*, *B2M*, *CUL3*, and *HLA-DRA*. All genes within the cluster were downregulated.

### Differential expression analysis of *in vitro* basal cell samples

**FIGURE 4:**
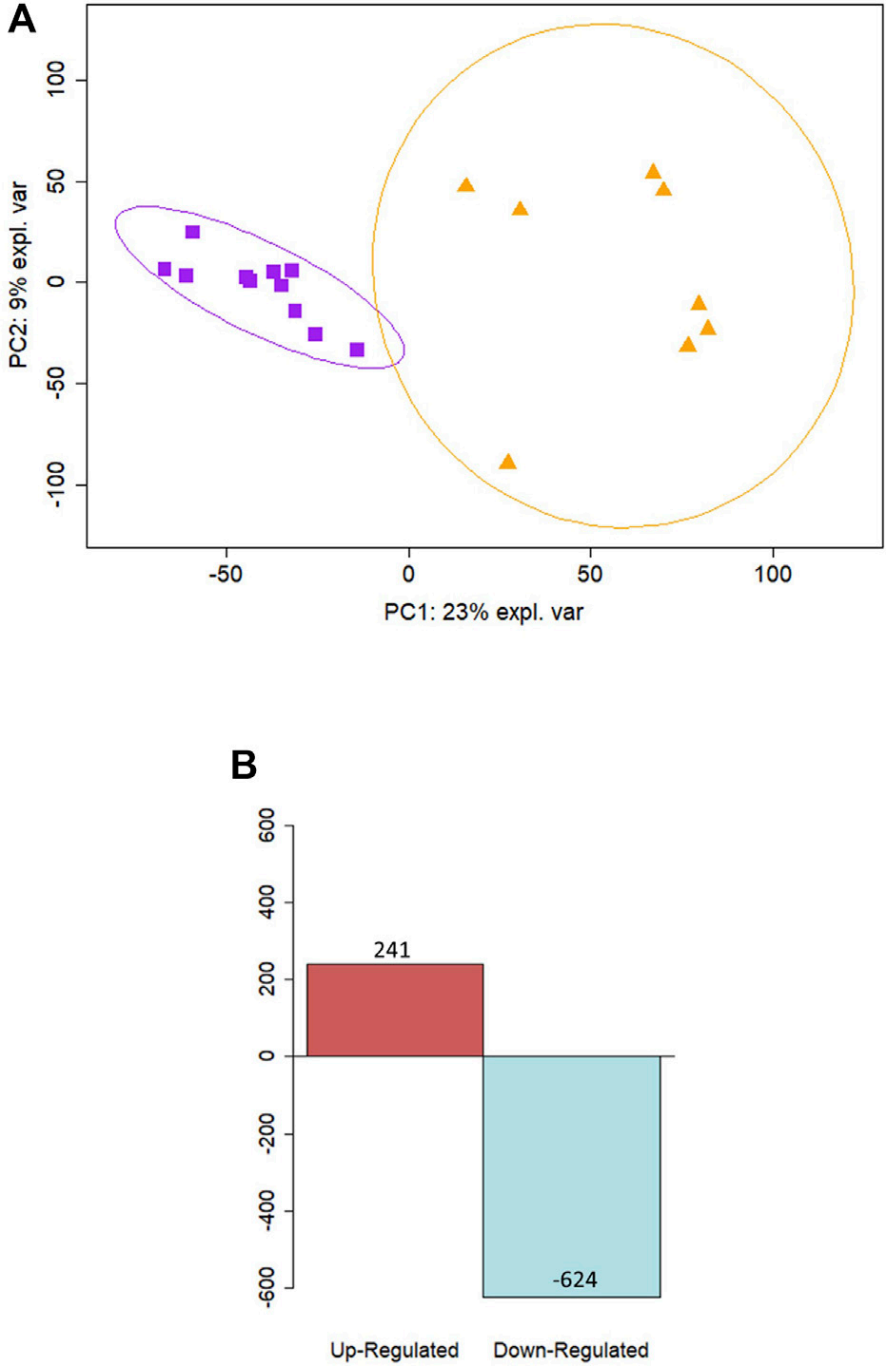
Differential expression analysis reveals distinct separation of term and preterm basal cell samples. **(A)** PCA analysis of *in vitro* samples reveal separation along the PC1 component between preterm samples (n = 11) shown in purple squares and term samples (n = 5) in orange triangles. **(B)** Differential expression analysis revealed 241 upregulated genes and 624 downregulated in preterm *in vitro* samples compared to term controls.

*In vitro* samples were assessed to provide a targeted analysis of the basal cell population. Gene profiles detected in the *ex vivo* and *in vitro* populations were similar, with 9,452 genes of the 13,596 genes detected present in both sample types. The average gene expression was also similar between the *ex vivo* and *in vitro* samples, with a statistically significant correlation (r = 0.53, *p* < 0.001). As with *ex vivo* samples, PCA plots showed distinct clustering between term and preterm samples (Figure 4A). Genes contributing maximally to separation along the first component included *RICTOR*, *ZFR*, *LRRC40*, *PAPOLA*, *BIRC2*, *PPIL4*, *SNIP1*, *TRIP11*, *HAUS3*, and *GPATCH2.* The basal cell population showed 865 differentially modulated genes, of which 241 were upregulated and 624 were downregulated in the preterm cohort compared to term controls (Figure 4B). The top 10 up and downregulated genes are outlined in Table 3. Many of the differentially expressed genes were again associated with innate immunity, including those detected in the *ex vivo* samples such as *IDO1*, *TLR2*, *CD36*, *IL1R1*, *IRF1*, and *PSMB8*. In total, nine genes were significantly upregulated in both *ex vivo* and *in vitro* samples, and 69 genes were significantly downregulated in both *ex vivo* and *in vitro* samples. A full list of differentially expressed protein-coding genes is available in Supplementary File S1.

**TABLE 3:** The top differentially expressed genes of *in vitro* cultures of preterm samples compared to term samples. The top 10 up and downregulated genes (ranked by fold-change) are displayed.

Upregulated			Downregulated		
SYMBOL	log Fold Change	Adjusted *p*-value	SYMBOL	log Fold Change	Adjusted *p*-value
*CRCT1*	5.20	0.001	*TNFRSF6B*	−4.72	0.001
*SBSN*	4.47	0.011	*BPIFB1*	−4.89	<0.001
*LINC00707*	3.49	0.018	*STC1*	−4.69	<0.001
*SPRR2D*	3.30	0.005	*COL1A2*	−4.56	0.017
*KLK12*	3.23	0.042	*RARRES1*	−4.38	<0.001
*KRT4*	3.08	0.038	*CAPN13*	−4.25	<0.001
*CAPN1-AS1*	3.07	0.001	*BPIFA1*	−4.11	<0.001
*CALML5*	3.04	0.021	*CF1*	−4.08	<0.001
*SULT2B1*	2.93	0.001	*BPIFA2*	−4.00	<0.001
*RAET1L*	2.87	<0.001	*MMP7*	−3.95	<0.001

**FIGURE 5:**
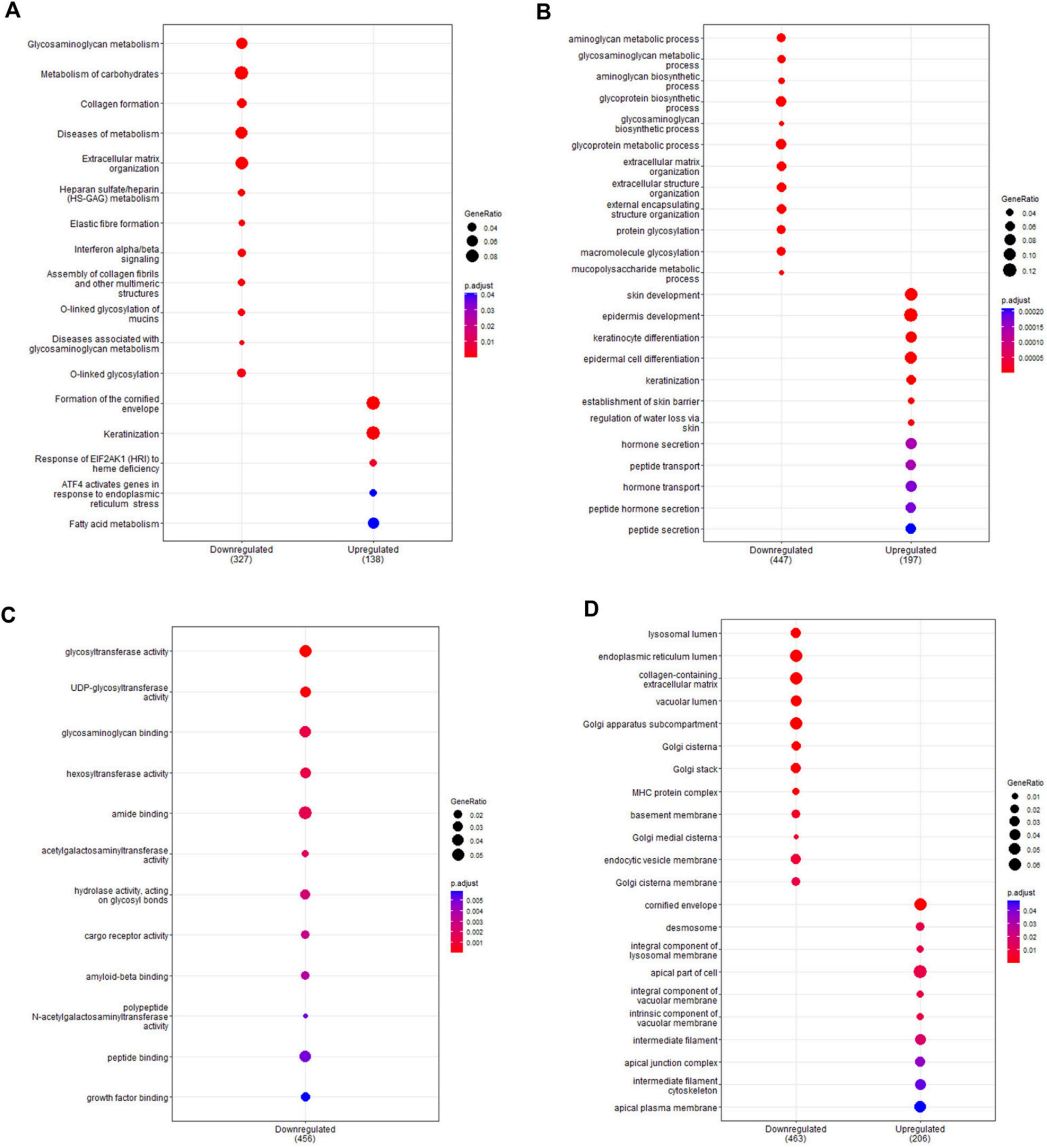
Pathway enrichment analysis of *in vitro* samples. Pathway enrichment analysis was performed on differentially expressed genes detected between *in vitro* term (n = 5) and preterm (n = 11) samples using the Reactome and clusterProfiler packages. Enrichment was performed using the Reactome database **(A)** and the Gene Ontology database for biological processes **(B)**, molecular function **(C)** and cell components **(D)**. The top 10 up and downregulated pathways are shown for each pathway analysis. The gene ratio (number of differentially expressed genes over the total pathway genes) is represented by the size of the circle, with the colour of the circle denoting the significance level of the *p*-value.

As with the *ex vivo* samples, analysis of functional gene sets within cultured basal epithelial cells identified numerous enriched pathways (Figure 5; full list in Supplementary File S2). The top pathways are presented across the Reactome (Figure 5A) and Gene Ontology databases for biological processes (Figure 5B), molecular function (Figure 5C) and cell component (Figure 5D). Top enriched pathways occurring multiple times in downregulated genes included interferon signalling (R-HSA-909733, R-HSA-913531, GO:0034341), elastic fibre formation (R-HSA-1566948, GO:0048251), extracellular matrix organisation (R-HSA-1474244, R-HSA-1474228, GO:0030198, GO:0043062, GO:0085029, GO:1903055, GO:0005201, GO:0030020, GO:0062023) and glycosylation activity (R-HSA-913709, R-HSA-5173105, R-HSA-3781865, GO:0016757, GO:0008194, GO:0016798, GO:0004553, GO:0006486, GO:0043413 GO:0070085, GO:0006493). In upregulated genes, top pathways included keratinization (R-HSA-6805567, GO:0030216, GO:0031424) cornification (R-HAS-6809371, GO:0001533) and peptide transport activity (GO:0015833, GO:0090087). A total of 84 enriched pathways were identified as common between *ex vivo* and *in vitro* samples, with repeating themes including interferon signalling, glycosylation, antigen processing and protein metabolism (full list in Supplementary File S2). As expected, pathways involving cilia function were not enriched in basal cell samples. Network analysis again revealed an abundance of immune related genes in the primary network (Supplementary Figure S2A) with functional clustering detecting a smaller cluster of interferon-related genes (Figure 2B). Genes identified within this cluster were *IRF1*, *TAP2*, *HLA-A*, *HLA-B*, *B2M*, *PSMB8*, *IF16*, *IFITM1*, *IRF5*, *KPNA1*, *KEAP1*, *CAT*, *ABL1*, *GNS*, and *GBP2*, with *IRF1* acting as the main hub gene. All genes within the cluster were downregulated in preterm cells except for *IRF5*, *TAP2*, and *KPNA* which were upregulated.

### Corroboration of interferon pathways

**FIGURE 6:**
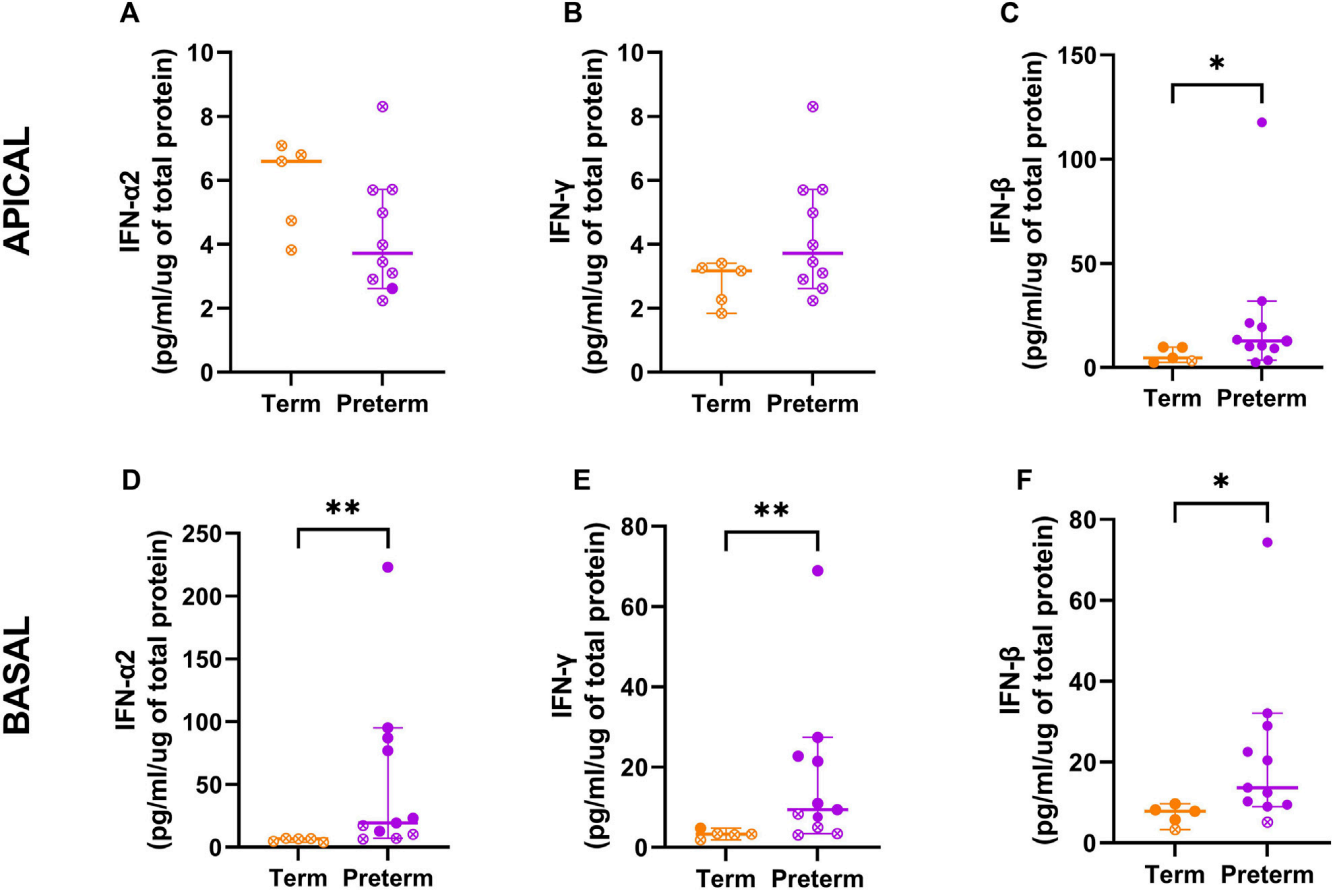
Interferon concentrations in cell culture supernatants from term and preterm participants. To validate enrichment of interferon pathways, the protein concentration of IFN-α2, IFN-γ and IFN-β were measured within cultured cell supernatants from the apical **(A–C)** and basolateral **(D–F)** compartments. Apical and basolateral concentrations of IFN-β were significantly higher in cells from preterm participants (n = 11) compared to term controls (n = 5). Concentrations of IFN-α2 and IFN-γ were also significantly elevated in basolateral supernatants from preterm participants. Data are shown as median with interquartile range and statistical differences between the term and preterm cohort were determined using the Mann-Whitney *U* test. Open symbols denote where measured concentrations were below detection limits and half of the lowest standard was used to calculate the cytokine release per µg of protein.

Both the *ex vivo* and basal cell samples identified enrichment in interferon signalling pathways as a major signature, and network analysis further identified interferon related modules. To corroborate these sequencing results, protein concentration of IFN-α2, IFN-γ, and IFN-β were measured in cell culture supernatants (Figure 6). In apical supernatants, representative of interferon release into the airways, IFN-α2 and IFN-γ were below measurable concentrations for all term and preterm samples (Figures 6A, B). IFN-β was measurable in all but one term sample and showed significantly elevated concentrations in preterm samples (Figure 6C; median [IQR]: 4.6 [6.9] vs. 12.8 [12.2] pg/ml/µg protein; *p* = 0.04). In the basolateral supernatant, representative of systemic release, IFN-α2 production was below measurable limits in term samples but was measurable in 7/11 preterm samples (Figure 6D; 6.6 [2.7] vs. 19.4 [76.7] pg/ml/µg protein, *p* = 0.03). IFN-γ production was measurable in one term and seven preterm samples, with concentrations significantly higher in preterm samples (Figure 6E; 3.3 [1.5] vs. 9.4 [17.7] pg/ml/µg protein; *p* = 0.01). Production of IFN-β was significantly higher in preterm supernatant, although one term and one preterm sample were below detection limits (Figure 6F; 7.8 [2.5] vs. 13.6 [19.5] pg/ml/µg protein, *p* = 0.01).

## Discussion

There is growing recognition of the role the airway epithelium plays in the development of chronic respiratory disease. However, there is a paucity of transcriptomic data to assess potential mechanisms within the airway epithelium in those born preterm. The present study aimed to assess if a distinct transcriptomic signature is detectable in nasal epithelial cells from individuals born preterm. Further, we aimed to assess the validity of an *in vitro* model to retain any transcriptomic signatures identified in those born preterm. These data show a distinct transcriptomic profile in the nasal airway epithelium of preterm birth survivors approximately 1 year after birth compared with term-born children. This distinct profile was detected in samples taken directly from the nasal cavity and in established monolayer primary cell cultures. We are the first to show that there is altered nasal airway epithelial gene expression in preterm birth survivors outside of the neonatal period. These findings suggest that preterm birth survivors have changes in their nasal airway epithelium that are detectable in the absence of the acute trauma and interventions associated with intensive care. Such changes may consequently provide insight into potential mechanisms of respiratory disease and targets for therapeutic intervention in those born preterm.

Analysis of the differentially expressed genes between term and preterm nasal epithelial cells found an over-representation of immune-related pathways, particularly those relating to innate immunity. Our data found that alpha, beta and gamma interferon signalling pathways were all overrepresented. Specifically, we found mRNA expression of interferon genes were low in the preterm group, whilst the protein concentrations of IFN-α, IFN-β, and IFN-γ were elevated in preterm samples. The discrepancy between mRNA expression and protein concentrations likely explains why some interferon stimulated genes, such as *TRIM56*, were upregulated in the preterm samples. Altered protein activity, which was not directly assessed in our study, may also contribute towards this discrepancy. Interferon production plays a fundamental role in orchestrating the anti-viral defence (Samuel, 2001; Katze et al., 2002; Sadler and Williams, 2008), which is intriguing given viral infection is the leading cause of hospital re-admission in those born preterm (Hong et al., 2016; Simpson et al., 2018). However, the known associations between interferons and infection also makes it important to consider that we did not screen the epithelial samples for the presence of virus or bacterial pathogens. Whilst participants were asymptomatic at the time of sample collection and cultures showed no visible sign of infection, it is possible that any differences in interferon profiles reflect recent infection rather than an intrinsic difference.

Although it remains uncertain if asymptomatic or recent infection altered the interferon profile in this study, previous studies have hinted at disrupted interferon profiles after preterm birth. Transcriptomic analysis by Shui et al. (2021), using airway epithelial cells collected from neonates shortly after birth, identified enrichment of interferon signalling pathways in those born preterm. Increased interferon protein concentrations have also been reported in saliva samples collected from preterm infants shortly after delivery compared to term infants (Sesso et al., 2014). Here, it was found that interferon concentrations continued to increase in those born preterm, with concentrations significantly higher in saliva samples collected at 3 months of age compared to matched neonatal samples (Sesso et al., 2014). A separate study found increased IFN-γ concentrations in the tracheal aspirate of preterm neonates within the first 48 h of life were associated with an increased risk of the infant developing bronchopulmonary dysplasia (Sesso et al., 2014). Together, these findings suggest that interferon concentrations are indeed altered by preterm birth and may contribute to poor respiratory outcomes, although the exact functional implications of this remains to be assessed. Similarly, it remains unknown if interferon concentrations remain elevated throughout childhood.

It is unknown if gene expression changes with age in those born preterm, which limits the ability to interpret the findings within this study. The immune system continues developing throughout life (Simon et al., 2015), and the differential expression of immune genes may consequently reflect delayed development that will correct over time. It is therefore also possible that the age difference between the term and preterm participants in this study could be influencing the transcriptomic results. However, age was included into the model as a co-variate to minimise the effect of age-related differences and it has previously been reported that the healthy nasal transcriptome was over 80% conserved between young children and teenagers (Chu et al., 2016). Genes that they identified as having the most variable expression depending on age, including *PROK2* and *IL1B* (Chu et al., 2016), were not differentially expressed in our cohorts. It is therefore unlikely that age at sample collection is a significant driver in the differences observed between the term and preterm samples. However, future work should consider repeating transcriptomic analysis in a longitudinal and gender-balanced cohort to identify if altered gene expression continues throughout childhood or corrects with increasing age. Experimental validation of the sample’s cellular composition or the use of single-cell sequencing should also be considered in future studies, given deconvolution estimates of the *ex vivo* samples suggests that the preterm population had higher proportions of ciliated cells. Although this does not impact the findings from the cultured basal cells, different cell populations would impact the transcriptomic signature of *ex vivo* samples.

The secondary outcome of this study was to identify if the transcriptome was conserved in cultured epithelial cells collected from those born preterm. A primary cell culture model that can retain an *ex vivo* signature allows for future mechanistic work and drug intervention studies to provide disease-specific insight. We saw significant overlap between the genes detected in *ex vivo* samples and the cultured basal cells, as well as overlap between the overexpressed pathways. Whilst exact gene expression levels varied, these data indicate that many of the *ex vivo* characteristics of the preterm airway epithelium are retained in an *in vitro* model. Nasal samples are considered a suitable surrogate for the lower airway under the unified airway hypothesis, with over 90% of the gene expression signature being conserved between the upper and lower airway (Kicic et al., 2020). The use of nasal samples is also beneficial, given samples can be collected without the need for intubation and general anaesthetic (which is required for sampling of the lower airway). Consequently, using nasal primary epithelial cells to establish cell-culture models is likely to be a suitable surrogate to study the preterm airway epithelium and would be invaluable to explore the functional impact of dysregulated gene expression and pathways identified within this analysis. However, as differences can still exist between nasal and bronchial cells (Rodenburg et al., 2023), future studies should consider doing a direct comparison of nasal and bronchial cells in a preterm population to confirm the generalisability of the nasal epithelium as a model.

In conclusion, this study has revealed that the nasal epithelial cells exhibit a distinct transcriptomic signature 1 year after preterm birth. Immune-related pathways are over-represented in the preterm nasal airway epithelium, with one third of differentially expressed genes identified as having a role in immune regulation or activation. Interferon production was significantly altered, with the elevated concentrations found in this study reflecting findings from previous research. However, it is unclear whether this transcriptomic signature will persist throughout childhood. Additional studies to investigate the functional epithelial and immunological defects in preterm birth survivors beyond the neonatal intensive care unit would provide valuable insight. Identifying and working to understand the mechanistic drivers and pathways of chronic lung disease will be imperative to improve long-term clinical outcomes of those born preterm.

## Data Availability

The data presented in the study are deposited in the Gene Expression Omnibus repository, accession number GSE272308.

## Supplementary material

SUPPLEMENTARY FIGURE S1 Network analysis of ex vivo samples*.* Network analysis was performed on differentially expressed genes between preterm and term participants’ *ex vivo* samples. **(A)** The InnateDB gene list was used to highlight in purple the number of genes related to immunity within the primary network. **(B)** A module of interferon-related genes was identified within the network of immune-related genes. Downregulated genes were coloured blue, with all genes within the module being downregulated.

SUPPLEMENTARY FIGURE S2 Network analysis of in vitro samples*.* Network analysis was performed on differentially expressed genes between preterm and term *in vi*tro samples. **(A)** The InnateDB gene list was used to highlight genes related to immunity from the primary network (shown in purple). **(B)** A module of interferon-related genes was identified within the network of immune-related genes, with upregulated genes shown in red and downregulated genes shown in blue.

SUPPLEMENTARY TABLE S1 Demographic data for the entire preterm cohort (PIFCO) that underwent nasal brushings. No significant differences were observed in the demographic data from the wider cohort compared to the preterm samples used for the *ex vivo* and *in vitro* sequencing.
