# Supplementary figures and images for: Transcriptomic analysis of primary nasal epithelial cells reveals altered interferon signalling in preterm birth survivors at one year of age
Source: Front Cell Dev Biol. 2024 Jul 24;12:1399005. doi: 10.3389/fcell.2024.1399005 (PMC11303191; doi:10.3389/fcell.2024.1399005)

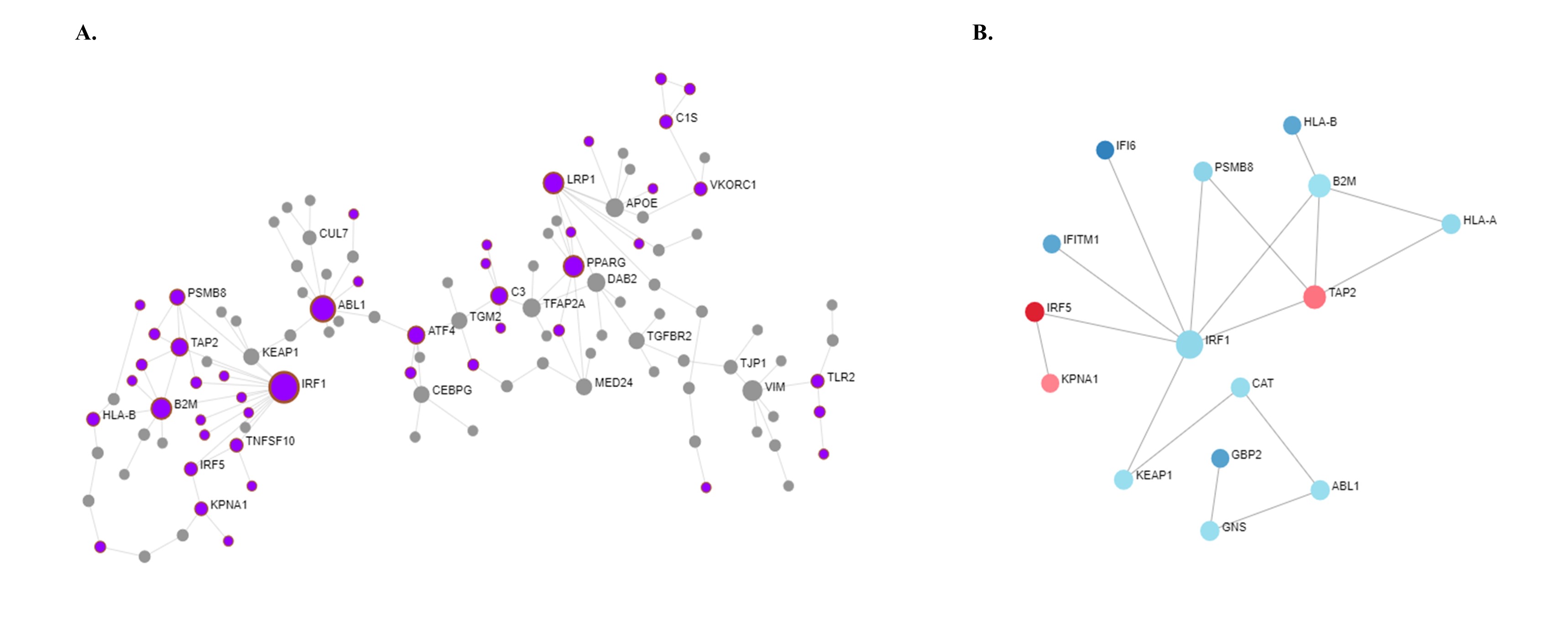

Supplement: Supplementary file 2 [file Image3.JPEG]

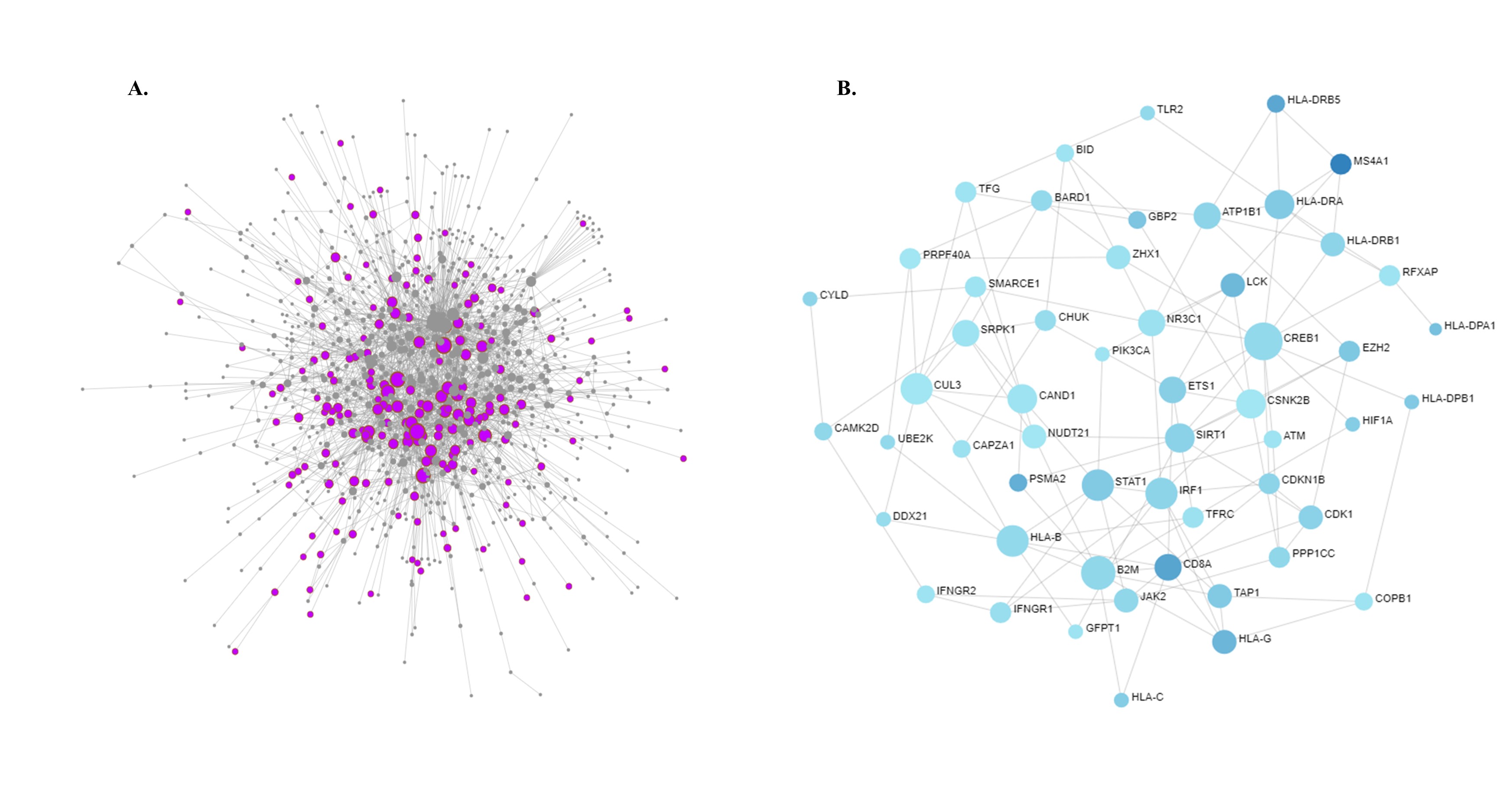

Supplement: Supplementary file 3 [file Image1.JPEG]

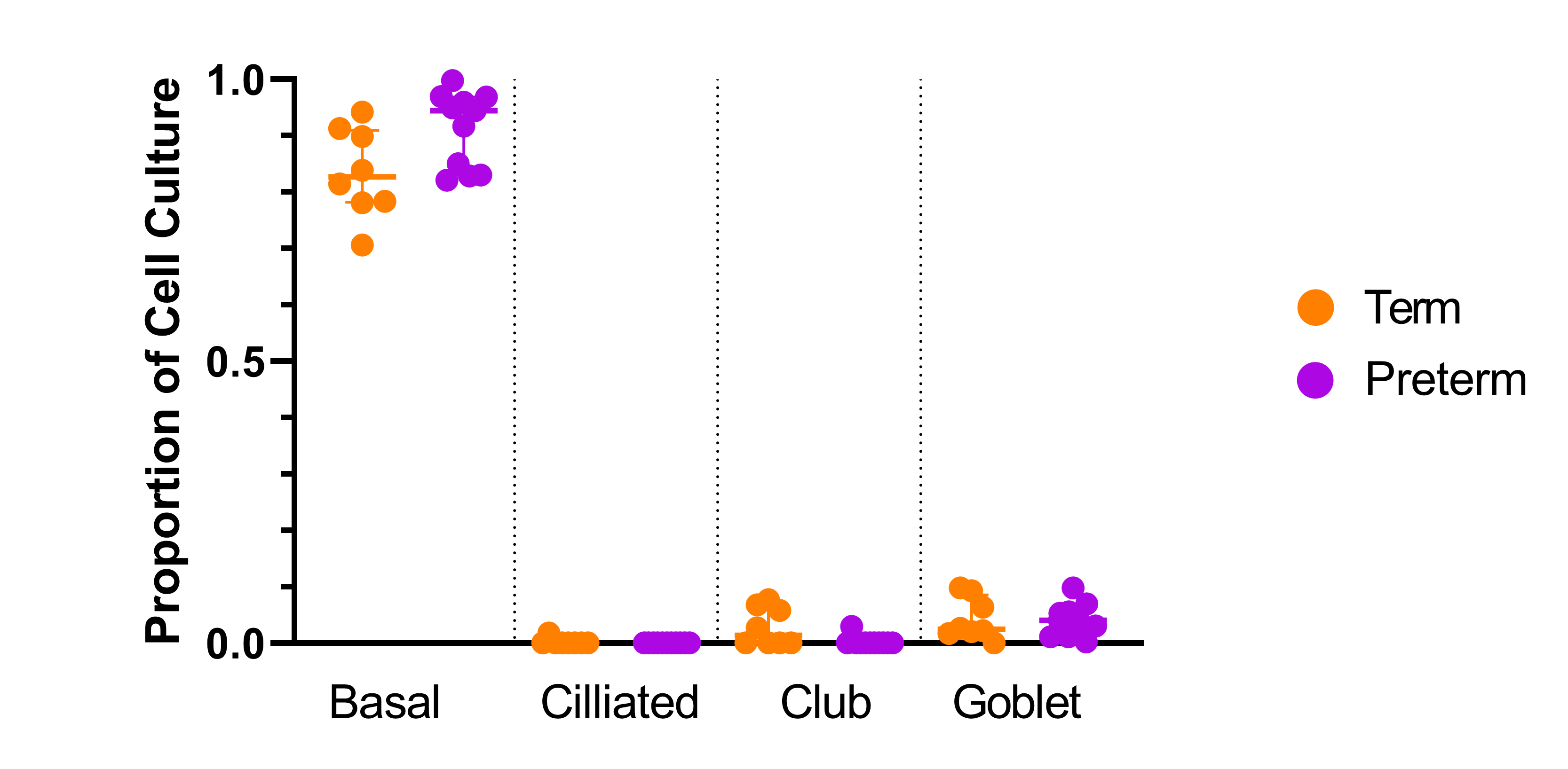

Supplement: Supplementary file 4 [file Image2.TIF]
