# Supplementary material for: Transcriptomic analysis of primary nasal epithelial cells reveals altered interferon signalling in preterm birth survivors at one year of age
Source: Front Cell Dev Biol. 2024 Jul 24;12:1399005. doi: 10.3389/fcell.2024.1399005 (PMC11303191; doi:10.3389/fcell.2024.1399005)
Supplement: Supplementary file 5 [file Table3.DOCX]

**Supplementary Table 1. Demographic data for the entire preterm cohort (PIFCO) that underwent nasal brushings.** No significant differences were observed in the demographic data from the wider cohort compared to the preterm samples used for the *ex vivo* and *in vitro* sequencing.

|  | **Full Cohort** | ***Ex vivo* Samples** | ***In vitro***  **Samples** |
| --- | --- | --- | --- |
| **Sample (n male)** | 35 (28) | 9 (9) | 11 (9) |
| **Age at Brushing**  **(Actual Years)** | 1.42  ± 0.99 | 1.45  ± 0.10 | 1.40  ± 0.12 |
| **Gestational Age (Weeks)** | 27.7  ± 2.35 | 27.2  ± 2.81 | 28.32  ± 1.95 |
| **Bronchopulmonary Dysplasia (%)** | 15 (43%) | 4 (44%) | 4 (36%) |
| **Birthweight Z-Score** | 0.03  ± 0.91 | -0.33  ± 0.78 | -0.12  ± 0.89 |
| **Total Hours Oxygen** | 198 [1582] | 61 [1597] | 59 [1203] |
| **Total Hours Respiratory Support (Ventilation, CPAP and HHF)** | 1323  ± 863 | 1401  ± 895 | 1313  ± 729 |
